# Association Between Occlusal Interferences, Temporomandibular Joint Dysfunction, and Bruxism in Romanian Adults

**DOI:** 10.3390/jcm14165612

**Published:** 2025-08-08

**Authors:** Ioana Elena Lile, Tareq Hajaj, George Dumitru Constantin, Serban Talpos Niculescu, Diana Marian, Otilia Stana, Cristian Zaharia, Ioana Veja

**Affiliations:** 1 Department of Dental Medicine, Faculty of Dentistry, “Vasile Goldis” Western University of Arad, 310025 Arad, Romania; lile.ioana@uvvg.ro (I.E.L.); marian.diana@uvvg.ro (D.M.); stana.otilia@uvvg.ro (O.S.); veja.ioana@uvvg.ro (I.V.); 2 Department of Prostheses Technology and Dental Materials, Faculty of Dentistry, “Victor Babes” University of Medicine and Pharmacy, 2 Eftimie Murgu Sq., 300041 Timisoara, Romania; tareq.hajaj@umft.ro (T.H.); cristian.zaharia@umft.ro (C.Z.); 3 Research Center in Dental Medicine Using Conventional and Alternative Technologies, Faculty of Dental Medicine, “Victor Babes” University of Medicine and Pharmacy of Timisoara, 9 Revolutiei 1989 Ave., 300070 Timisoara, Romania; 4 Discipline of Clinical Practical Skills, Department I Nursing, Faculty of Medicine, Victor Babes University of Medicine and Pharmacy, 300041 Timisoara, Romania; 5 Department of Oral and Maxillofacial Surgery, Faculty of Dentistry, “Victor Babes” University of Medicine and Pharmacy, 2 Eftimie Murgu Sq., 300041 Timisoara, Romania

**Keywords:** bruxism, sleep bruxism, awake bruxism, malocclusion, temporomandibular joint disorders, parafunctional habits, psychological stress, sleep apnea syndromes, self-reported questionnaire, cross-sectional analysis

## Abstract

**Background:** Bruxism is a common parafunctional activity involving repetitive jaw muscle movements during wakefulness (awake bruxism) or sleep (sleep bruxism). While its multifactorial etiology is widely accepted, the roles of occlusal interferences, temporomandibular joint (TMJ) dysfunction, stress, and sleep-related breathing disturbances remain under investigation. **Objectives**: This cross-sectional study evaluated associations between bruxism and occlusal discrepancies, TMJ symptoms, stress, and sleep-related variables (snoring and obstructive sleep apnea, OSA) in Romanian adults. **Methods**: Ninety-eight adults (mean age: 30.4 ± 8.9 years) completed a structured questionnaire reviewed for content validity and pilot-tested for clarity but not formally validated. Participants were categorized into bruxism (*n* = 51) and control (*n* = 47) groups. Variables assessed included bruxism type, TMJ symptoms, occlusal interferences, stress, snoring, OSA, and parafunctional habits. Bivariate and multivariate logistic regression analyses were conducted. **Results**: Sleep bruxism was predominant (88%), with 59% classified as sleep-only bruxers. Occlusal discrepancies (46% vs. 14%, *p* < 0.001), TMJ symptoms (joint pain: 38% vs. 8%; fatigue: 44% vs. 10%), and habitual snoring (60% vs. 22%, *p* < 0.001) were significantly more common among bruxers. No significant difference was observed in OSA prevalence. Independent predictors of bruxism included occlusal interferences (adjusted OR = 4.7, *p* = 0.009), TMJ symptoms (adjusted OR = 6.5, *p* = 0.002), and habitual snoring (adjusted OR = 4.1, *p* = 0.016). **Conclusions**: Bruxism was significantly associated with occlusal interferences, TMJ dysfunction, and habitual snoring, supporting the need for multifactorial assessment and integrated clinical management. Limitations: This study relied on self-reported bruxism classification and a non-validated questionnaire instrument, which may limit generalizability and diagnostic accuracy.

## 1. Introduction

Bruxism is a prevalent oral parafunctional behavior characterized by repetitive jaw-muscle activity, including clenching, grinding, bracing, or thrusting of the mandible. It is typically categorized as either sleep bruxism (SB), which occurs during sleep, or awake bruxism (AB), which occurs during wakefulness. Recent consensus reports emphasize that bruxism is a centrally regulated behavior influenced by multiple biopsychosocial factors, with occlusion playing a contributory but not primary role [1,2,3]. This perspective is supported by recent systematic reviews that recommend cautious interpretation of occlusal factors in bruxism etiology [4,5].

Though traditionally considered a benign habit, bruxism has been associated with multiple clinical consequences, including abnormal tooth wear, restoration failure, tooth mobility, orofacial pain, and compromised masticatory efficiency. These outcomes may also impair quality of life and increase the risk of temporomandibular joint (TMJ) dysfunction and sleep disturbances [4,5].

Sleep bruxism has been classified by the International Classification of Sleep Disorders as a sleep-related movement disorder, believed to originate in response to sleep arousals [6], while awake bruxism is thought to be more closely associated with emotional states and behavioral patterns such as stress, anxiety, and concentration [7]. Epidemiological data indicate regional variability in bruxism prevalence, with higher rates observed in Europe and the Americas [8]. According to the International Classification of Sleep Disorders, 3rd edition (ICSD-3), sleep disorders are categorized into six major groups: (1) insomnia, (2) sleep-related breathing disorders, (3) central disorders of hypersomnolence, (4) circadian rhythm sleep-wake disorders, (5) parasomnias, and (6) sleep-related movement disorders [7]. Bruxism is classified under sleep-related movement disorders, while obstructive sleep apnea (OSA) falls under sleep-related breathing disorders [2,9,10]. Both conditions have been associated with disturbances in the stomatognathic system and may co-occur or share overlapping risk factors. In addition to OSA, central sleep apnea (CSA) is another major category of sleep-disordered breathing. Unlike OSA, which results from upper airway obstruction, CSA is characterized by a lack of respiratory effort due to instability in the respiratory control centers of the brain. While its direct implications on the stomatognathic system are less frequently studied, CSA may contribute to fatigue, arousals, or orofacial tension patterns that potentially influence conditions like bruxism. Recognizing the distinct pathophysiological profiles of these disorders is essential for accurately interpreting their associations with parafunctional behaviors.

The contribution of dental occlusion to bruxism has been long debated. Earlier studies suggested that premature contacts or occlusal interferences might initiate bruxism or exacerbate its severity [3]. However, subsequent research has largely refuted a direct causal relationship, emphasizing central nervous system mechanisms as the primary drivers of the condition [9,11]. Today, occlusion is considered a potential, but not primary, factor within a multifactorial etiology involving stress, sleep arousals, and genetic predisposition [2,4].

TMJ dysfunction is frequently reported among individuals with bruxism, likely due to sustained mechanical loading of the joint and surrounding musculature. Bruxism may lead to orofacial pain, joint sounds, limited jaw mobility, and morning jaw stiffness, and evidence supports a strong comorbidity with temporomandibular disorders (TMD) [12,13,14,15]. Whether TMJ symptoms are a cause or consequence of bruxism remains unclear, but their clinical overlap underscores the importance of dual assessment.

Psychological stress, particularly chronic stress, has been consistently linked to both forms of bruxism. It is believed to contribute via sympathetic nervous system activation, heightened muscle tone, and disturbed sleep patterns [6,16,17]. Although this relationship is well established, many studies rely solely on subjective stress measures, which may introduce bias.

Recent studies have also examined possible connections between bruxism and sleep-disordered breathing, particularly habitual snoring and OSA. While some suggest that micro-arousals from OSA may trigger bruxism as a compensatory mechanism to reopen the airway [18], others have found no significant independent association after adjusting for shared risk factors such as obesity or smoking [19,20].

Despite increasing recognition of bruxism’s multifactorial nature, much of the existing research is based on subjective reports or non-standardized instruments, limiting comparability and generalizability [1,8,9,14]. Objective diagnostic tools such as polysomnography, electromyography, intraoral scanning, or TMJ imaging are not always accessible in large-scale studies. Thus, questionnaire-based data—while valuable—should be interpreted with appropriate caution.

Given these constraints, the use of a self-reported instrument in the present study was a pragmatic approach to allow for broad data collection in a general population sample. Although not a replacement for objective diagnostic tools, self-report methods remain widely used in bruxism research and can yield meaningful insights when carefully constructed and interpreted within their methodological limitations. Our questionnaire was developed with input from domain experts, reviewed for content validity, and pilot-tested to ensure clarity and thematic coverage. The results of the pilot study were not published separately.

To address these gaps, the present study investigated the association between bruxism and key potential contributors—occlusal discrepancies, TMJ-related symptoms, stress, and sleep-related breathing disturbances—in a Romanian adult population. Although based on a self-report instrument, the questionnaire was developed with expert input and pilot-tested to ensure thematic breadth and clarity. Our aim was to provide a preliminary but integrative view of bruxism’s complex etiology in the general population, which may guide future research using objective assessments.

This study focused on Romanian adults due to the current lack of population-based data on bruxism and its potential correlates in this demographic. Given logistical and resource constraints, a self-reported method was selected to enable wide recruitment and preliminary exploration of relevant behavioral and clinical patterns. Although this approach has inherent limitations in diagnostic precision, it is well suited for hypothesis-generating research and helps address regional gaps in the bruxism literature.

Given the multifactorial etiology of bruxism and the scarcity of population-based studies examining its associations with occlusal interferences, temporomandibular joint (TMJ) symptoms, and sleep-related factors, this study aimed to explore these relationships in a sample of Romanian adults. While self-reported data present inherent limitations, they allow for broad, scalable assessments in non-clinical settings and can generate valuable hypotheses for future research. This investigation contributes to the growing body of evidence on the behavioral and structural correlations of bruxism and highlights the need for integrated, interdisciplinary approaches to its assessment.

Despite its methodological limitations, this study offers several strengths. It contributes to the contemporary literature by adopting a multifactorial approach to bruxism assessment, integrating self-perceived occlusal, musculoskeletal, and sleep-related parameters within a single analytical framework. The use of a structured, content-validated, and pilot-tested questionnaire enabled broad data capture in a community sample, providing exploratory insights that may inform future research designs. Importantly, the findings emphasize the need for interdisciplinary investigation of bruxism-related complaints, which may have implications for both dental and sleep medicine practice.

Based on the current evidence suggesting multifactorial contributors to bruxism, we tested the null hypothesis that there are no significant associations between self-reported bruxism and occlusal interferences, TMJ symptoms, perceived stress, or sleep-related factors in Romanian adults.

## 2. Materials and Methods

### 2.1. Study Design and Participants

This cross-sectional observational study was conducted between March and June 2025 in accordance with the STROBE guidelines. This study was designed and reported in accordance with the STROBE checklist for cross-sectional studies. Efforts to mitigate potential sources of bias included anonymized data collection, the use of standardized survey items, and adjustments for key confounders in the multivariate analysis. Participants were recruited via an online questionnaire distributed through university and social media networks using a snowball sampling strategy. We acknowledge that snowball sampling via social media constitutes a non-probability sampling method and may introduce self-selection bias, potentially limiting the generalizability of the findings. This technique was chosen for its feasibility in recruiting a diverse adult sample during a defined study period and for its relevance in identifying individuals with specific behavioral traits such as bruxism. Eligible respondents were Romanian adults aged 18–60 years who provided electronic informed consent. The study protocol was approved by the Ethics Committee of the Vasile Goldiș Western University of Arad (Approval No. 17/19 March 2025), and all procedures complied with the Declaration of Helsinki. Anonymity and confidentiality were ensured.

This study was designed to explore potential associations between self-reported bruxism and contributing factors—including occlusal interferences, TMJ symptoms, stress, and sleep-related behaviors—in a general adult Romanian population. Given the lack of national data and limited access to objective diagnostic tools in large samples, a structured, self-reported approach was used to enable broad, hypothesis-generating analysis.

#### 2.1.1. Inclusion Criteria

Participants were eligible for inclusion if they met all of the following criteria:

- Aged 18 years or older
- Romanian nationality and residence
- Ability to understand and complete the questionnaire in Romanian
- Consent to participate voluntarily in the study

#### 2.1.2. Exclusion Criteria

Participants were excluded if they met any of the following criteria:

- Incomplete or inconsistent questionnaire responses
- Diagnosed psychiatric or neurological disorders that may affect perception or self-report accuracy (based on self-report)
- Previous maxillofacial surgery or conditions that could significantly alter occlusion or TMJ function

A total of 114 participants were enrolled; after exclusions, 98 complete responses were analyzed, 51 identified with bruxism and 47 without. The minimum required sample size (*n* = 46 per group) was calculated using Epidat 4.2 (α = 0.05, 80% power).

### 2.2. Survey Instrument and Variables

A 25-item structured questionnaire was developed by the research team to collect data relevant to the objectives of the study. The questionnaire was developed based on established bruxism assessment tools such as TMJ-related surveys and published observational studies in dental and behavioral sciences. Although the instrument was not previously validated, it underwent face and content validation by two subject-matter experts in prosthodontics and orofacial pain. A pilot study with five volunteers tested clarity, completion time, and item comprehensibility, leading to minor refinements to enhance clarity and consistency before distributing it to the full study sample. A structured questionnaire reviewed for content validity and pilot-tested for clarity was used.

Although the questionnaire was developed with domain expert input and pilot-tested for clarity, it was not formally validated through psychometric analysis. The internal consistency of items was not assessed, as the survey comprised distinct domains rather than coherent subscales. For perceived stress, only two items adapted from the PSS were included to minimize participant fatigue. However, we acknowledge that the full PSS-10 or PSS-14 is recommended for more robust assessment in future studies. The questionnaire was organized into four thematic sections: demographic data, bruxism characteristics, occlusal and TMJ-related factors, and behavioral or systemic variables. The key domains assessed are detailed below:

- Bruxism Status: Participants were asked if they experience tooth clenching or grinding during sleep or while awake, using lay descriptions. Bruxism status was determined based on self-report of symptoms (e.g., jaw discomfort, grinding noises, fatigue), third-party observation (e.g., partner’s report), or prior professional diagnosis. Participants were categorized as having bruxism if they answered affirmatively or reported suggestive signs. Subtypes were classified as SB, awake bruxism (AB), or a combination of both (SB + AB).
- Occlusal Factors: Participants were asked whether a dentist had ever informed them of occlusal interferences, premature contacts, or malocclusion. They were also asked if recent dental restorations or malpositioned teeth were perceived to affect their bite. These self-reported occlusal issues were recorded as a composite variable representing the presence of occlusal interferences.
- TMJ Symptoms: Self-reported symptoms included jaw joint pain, joint noises (clicking, crepitus), and morning jaw stiffness or fatigue. Responses were dichotomized (yes/no) for each symptom, and the presence of any symptom was treated as indicative of TMJ dysfunction. Respondents were also asked if they had ever been diagnosed with a temporomandibular disorder (TMD) by a healthcare provider.
- Parafunctional Habits: Behavioral contributors such as nail biting, habitual gum chewing, or object chewing (e.g., pens, pencils) were assessed through yes/no questions. These behaviors were analyzed as potential confounding variables due to their known impact on masticatory activity and joint loading.
- Sleep and Respiratory Factors: Participants reported whether they or their bed partners observed habitual snoring. They were also asked whether they had been diagnosed with OSA or suspected of having it, based on a provided lay definition. Responses were categorized as none, suspected, or diagnosed OSA.
- Perceived Stress: Stress levels were evaluated using two adapted items from the Perceived Stress Scale (PSS), a widely used psychological instrument for measuring perceived stress developed by Cohen et al. [21] focused on daily life and work-related stress. Items were rated on a 5-point Likert scale (1 = strongly agree that life is stressful, 5 = strongly disagree). Lower scores indicated higher stress levels. These were treated as ordinal variables and also dichotomized for high-stress analyses (scores ≤ 2).
- Other Variables: Basic demographics included age (categorized: <20, 20–29, 30–39, 40–49, ≥50), gender (male/female), and occupational status (student, employed, unemployed). Bruxism participants were asked about treatment history (e.g., occlusal splint, medication, stress management) and perceived treatment effectiveness.

The questionnaire was developed specifically for this study based on existing literature and expert input, and it was pilot-tested for clarity and content validity. However, it was not subjected to formal psychometric validation or internal consistency analysis, which represents a methodological limitation. The questionnaire underwent content validation by a panel of five academic experts in dentistry and behavioral sciences. To assess clarity and usability, a pilot study was conducted over a two-week period using a convenience sample of 12 adult volunteers (aged 22–48) affiliated with the university. Eligible participants for the pilot study were fluent in Romanian, not involved in the main study, and willing to provide feedback. Respondents completed the questionnaire and offered comments on item clarity, relevance, and overall comprehension. Based on their suggestions, minor wording adjustments were made to improve the final version used in the main survey. The full questionnaire is provided in Supplementary Material S1.

### 2.3. Data Analysis

The minimum required sample size was calculated using *Epidat* version 4.2, assuming a confidence level of 95% (α = 0.05), a statistical power of 80%, and an expected effect size (OR ≥ 3.0) based on previously reported associations between bruxism and TMJ symptoms [16]. The sample size was calculated based on an expected odds ratio (OR) ≥ 3.0, consistent with previous studies that reported moderate-to-strong associations between bruxism and TMJ symptoms in non-randomized populations [16]. This assumption also aligns with effect size interpretation guidelines proposed by Chen et al. [22]. Effect size interpretation for odds ratios followed thresholds proposed by Chen et al. [22] (small = 1.68, medium = 3.47, large = 6.71) and used here for illustrative purposes. However, we acknowledge that such thresholds are not standard in clinical studies and should be interpreted within the broader clinical context. A minimum of 46 participants per group was required to detect a statistically significant difference.

Data were entered into MedCalc v20.110 (MedCalc Software Ltd., Ostend, Belgium). Categorical variables were coded as binary (yes = 1, no = 0), Likert-scale items were treated as ordinal, and age categories were assigned midpoint values where needed. Incomplete responses were excluded. Descriptive statistics were calculated for the full sample and by bruxism status. Group comparisons used chi-square tests for categorical variables and independent *t*-tests or Mann–Whitney U tests for continuous/ordinal data, based on normality. Group comparisons used chi-square tests for categorical variables. For continuous variables with a normal distribution (e.g., age), independent *t*-tests were applied. For ordinal variables and continuous variables without a normal distribution (e.g., perceived stress scores), Mann–Whitney U tests were used.

Associations between bruxism and key variables (occlusal interferences, TMJ symptoms, snoring, OSA, stress, oral habits) were tested using 2 × 2 contingency tables. Odds ratios (OR) and 95% confidence intervals (CI) were computed; Fisher’s exact test was used when applicable. A multivariate logistic regression model identified independent predictors of bruxism. Variables included occlusal interferences, TMJ symptoms (combined), high perceived stress, snoring, age, and gender. Adjusted odds ratios (aOR) and 95% CIs were reported. Model fit was assessed using the Hosmer–Lemeshow goodness-of-fit test and Nagelkerke’s R^2^ coefficient in accordance with standard recommendations for logistic regression evaluation [23].

Effect size interpretation for odds ratios (ORs) followed established thresholds: small (OR = 1.68), medium (OR = 3.47), and large (OR = 6.71) as suggested by Chen et al. [22].

Statistical significance was set at *p* < 0.05. No correction for multiple comparisons was applied due to the exploratory nature of some analyses.

Given the exploratory nature of the study and the limited sample size, no correction for multiple comparisons was applied. We acknowledge that this may increase the risk of type I errors and the results should be interpreted with caution.

### 2.4. Outcome Measures

The primary outcome was the association between bruxism and occlusal interferences. Secondary outcomes included relationships between bruxism and TMJ symptoms, perceived stress, snoring, OSA, and oral habits. Exploratory outcomes included self-reported treatment use and perceived effectiveness among bruxers.

## 3. Results

### 3.1. Participant Characteristics

Out of 124 individuals who accessed the online questionnaire, 98 completed all of the mandatory items, yielding a completion rate of 79%. Incomplete responses were excluded from analysis. No imputation was performed for missing data, as all retained records were complete. A total of 98 adults were included in the final analysis. The final sample consisted of 98 adult respondents: 51 participants were assigned to the bruxism group and 47 to the control group. Ages ranged from 18 to 59 years, with the 20–29 age range representing the largest subgroup (approximately 45% of all respondents), reflecting the snowball sampling via academic and professional networks. The mean age was 29.8 years (SD = 8.5) in the bruxism group and 31.0 years (SD = 9.2) in the control group. This difference was not statistically significant (*p* = 0.45), indicating a similar age distribution across groups.

Gender distribution was also identical, with 28 females (55%) and 23 males (45%) in each group (*p* = 1.0). Most participants were university students or employed professionals, and no statistically significant differences in occupational status were observed between groups. Participant demographics are summarized in Table 1.

Among those with bruxism, the majority (88%, *n* = 44) reported symptoms consistent with SB, while 40% (*n* = 20) indicated awake bruxism (AB). Combined SB and AB was reported in 28% of cases. The most common presentation was SB-only (59%). A clinical diagnosis of bruxism had been established in 41% of participants, while the remaining were self-reported based on characteristic behaviors and symptoms. Four individuals in the control group reported occasional clenching or grinding during periods of stress but were not diagnosed with bruxism; these were retained in the analysis due to low frequency and minimal clinical impact.

To provide additional context, we conducted descriptive subgroup analyses. SB was more frequently reported by males, while AB was slightly more common among females. Younger participants (<30 years) reported higher rates of SB, whereas AB appeared more evenly distributed across age groups. Differences in reported TMJ symptoms, stress perception, and snoring frequency were observed across gender and bruxism subtypes. However, due to the limited sample size, these findings were not tested for statistical significance and should be interpreted cautiously.

It should be noted that bruxism classification (sleep vs. awake) was based entirely on participants’ self-reports and, where applicable, third-party observations. The absence of clinical examination or polysomnographic confirmation limits the diagnostic accuracy and introduces the potential for misclassification.

It is important to note that bruxism subtypes were determined based on participants’ self-reported behaviors and, in some cases, third-party observations. This approach, while pragmatic for survey-based studies, relies on subjective recall and perceptions, which may limit the internal validity of subtype classification and introduce potential bias.

### 3.2. Occlusal Factors

Participants with bruxism reported a higher prevalence of occlusal disturbances compared to controls. These included perceived occlusal interferences or premature contacts. The association remained significant after adjusting for age, gender, stress, and snoring (Table 2).

### 3.3. TMJ Dysfunction Symptoms

TMJ-related symptoms—including joint pain, joint sounds, and morning jaw fatigue—were more frequently reported in the bruxism group. Overall, 60% of bruxers experienced at least one TMJ symptom, compared to 16% of controls (Table 2).

### 3.4. Perceived Stress

Although no statistically significant differences were found in mean stress scores, bruxism participants tended to report higher levels of perceived stress. Based on a 5-point Likert scale (where lower scores indicate greater stress), 44% of bruxers reported high daily stress (scores 1–2), compared to 30% of controls (U = 1045.5, Z = 1.39, *p* = 0.16). Similarly, high work- or study-related stress was noted by 36% of the bruxism group versus 24% of the controls (*p* = 0.20). Although high perceived stress appeared more frequent among participants with bruxism in univariate comparisons, this association was not statistically significant in the multivariate logistic regression model (*p* > 0.05). Therefore, its role as an independent predictor in this sample remains inconclusive. While these findings did not reach significance, they align with known psychological contributions to awake bruxism and were included in the multivariate analysis for completeness.

### 3.5. Sleep-Related Breathing

Habitual snoring was significantly more prevalent among participants with bruxism (60%) compared to those without (22%) (χ^2^(1) = 15.38, *p* = 0.00009; OR = 5.11, 95% CI: 2.1–12.4), indicating a possible co-occurrence. In contrast, no significant difference was observed for self-reported OSA, which was present in 22% of bruxers and 20% of controls (*p* = 0.82).

### 3.6. Parafunctional Habits and Dental Aesthetics

Parafunctional oral behaviors such as nail biting, chewing on pens, and excessive gum use were included as exploratory variables due to their potential—but not primary—relevance to bruxism. However, no statistically significant differences were observed between groups. Similarly, concerns related to dental aesthetics were presented for descriptive purposes only and were not central to the study’s aims. Parafunctional oral behaviors were reported at similar rates in both groups. For instance, nail biting was present in 28% of the bruxism group and 24% of controls (*p* > 0.3). Likewise, concerns related to dental aesthetics—such as dissatisfaction with tooth alignment or appearance—were reported by approximately 30% of respondents in each group (*p* = 0.95), suggesting no direct association with bruxism status.

Although parafunctional oral behaviors such as nail biting, pen chewing, and excessive gum use were reported in both groups, no statistically significant differences were observed. One possible explanation is that these habits may have occurred at a low frequency or intensity insufficient to generate sustained masticatory strain or to overlap with the pathophysiological mechanisms underlying bruxism. Additionally, some of these behaviors may be more transient or context-dependent (e.g., stress-induced), and thus less likely to manifest as chronic, repetitive activities comparable to bruxism. This interpretation is consistent with prior literature indicating that occasional parafunctional behaviors do not necessarily translate into sustained muscular hyperactivity or clinical consequences.

### 3.7. Treatment and Self-Perceived Outcomes

Among bruxism participants, 52% (*n* = 26) reported having received some form of treatment, most commonly custom-fitted occlusal splints. Of those, 88% reported perceived improvements in symptoms such as pain, function, and sleep quality. These outcomes, however, were based solely on self-reported experiences and were not validated through clinical follow-up or objective assessment. As such, they should be interpreted as exploratory observations rather than evidence of treatment efficacy.

### 3.8. Multivariate Predictors of Bruxism

Logistic regression identified three independent predictors of bruxism: occlusal interference (adjusted OR = 4.7, *p* = 0.009; medium-to-large effect), any TMJ-related symptom (adjusted OR = 6.5, *p* = 0.002; large effect), and habitual snoring (adjusted OR = 4.1, *p* = 0.016; medium effect). Other factors—such as high daily stress (adjusted OR = 2.2, *p* = 0.12), age (aOR = 0.98, *p* = 0.43), and male gender (aOR = 1.1, *p* = 0.85)—were not statistically significant. The model demonstrated good fit (Hosmer–Lemeshow *p* = 0.68) and explained 58% of the variance in bruxism presence (Nagelkerke R^2^ = 0.58) (Table 3). No significant interaction terms were identified.

## 4. Discussion

This study explored associations between self-reported bruxism and potential contributing factors in a sample of Romanian adults. Notably, participants with bruxism were more likely to report perceived occlusal interferences, TMJ-related symptoms, and habitual snoring. These findings underscore the multifactorial nature of bruxism while highlighting the need for cautious interpretation due to methodological constraints. Given the cross-sectional design of this study, causal inferences cannot be made. Although associations were observed between bruxism, TMJ-related symptoms, and occlusal interferences, the directionality of these relationships remains unclear. It is equally plausible that TMJ symptoms or occlusal discomfort may contribute to, rather than result from, bruxism. Longitudinal studies are necessary to clarify these temporal and causal dynamics. Individuals with bruxism were markedly more likely to report occlusal interferences, TMJ symptoms, and snoring, reinforcing a multifactorial model of bruxism pathogenesis that includes both peripheral and central contributors [1,2,5,10]. These findings highlight the multifaceted interaction among occlusal discrepancies, parafunctional activity, psychological stress, and airway-related disturbances in the pathophysiology of bruxism.

Several variables, including perceived stress, OSA, and parafunctional oral habits, did not show statistically significant associations with bruxism in this sample. These null findings should be interpreted cautiously. The limited number of stress items used (only two adapted from the PSS), reliance on self-reporting for OSA and habits, and the modest overall sample size may have reduced statistical power and construct validity. Additionally, the heterogeneity within subgroups may have obscured potential associations. Future studies employing validated tools, clinical screening, and larger stratified samples may better capture these complex relationships.

The role of occlusion in bruxism etiology has long been debated [8,9,24]. While previous reports emphasize the primacy of central nervous system mechanisms [8,25], others suggest that in certain individuals, occlusal interferences may trigger or exacerbate bruxism episodes as a neuromuscular response to disharmony [25,26]. In our study, occlusal discrepancies were independently associated with bruxism status in multivariate analysis. However, causality cannot be inferred from a cross-sectional design. While our findings indicate a potential association between perceived occlusal interferences and bruxism, these results are based on self-reports and should not be interpreted as conclusive evidence of clinical relevance. Future studies incorporating objective occlusal assessments are needed before recommending routine occlusal analysis in this context. Nonetheless, contemporary guidelines discourage irreversible occlusal adjustments unless supported by comprehensive diagnostic findings [24]. While some associations yielded moderate-to-high odds ratios, these effect sizes should be interpreted with caution. In the absence of objective clinical validation and given the exploratory nature of the study, the clinical meaningfulness of these associations remains uncertain. Future research is needed to determine whether these observed magnitudes translate into actionable clinical insights.

TMJ symptoms were significantly more prevalent among bruxers in our sample, particularly joint pain, joint sounds, and morning jaw fatigue. These findings are consistent with previous studies showing a strong overlap between bruxism and temporomandibular disorders (TMD), often attributed to chronic mechanical overload of the joint and masticatory musculature [12,27,28]. Although the direction of this relationship remains unclear, participant responses frequently indicated that bruxism preceded the onset of TMJ discomfort. This supports the hypothesis that bruxism can act as a precipitating factor in TMD development in susceptible individuals [12].

Psychological stress is widely acknowledged as a key driver of awake bruxism and a contributing factor to sleep bruxism [2,16,29]. While average stress scores did not differ significantly between groups in our study, a higher proportion of bruxers reported elevated perceived stress levels. However, stress did not emerge as an independent predictor in multivariate modeling, possibly due to the limited single-item stress scale used. More nuanced assessment using validated instruments is recommended in future research.

The observed association between bruxism and habitual snoring—but not with OSA—corresponds with findings from recent systematic reviews [30]. It is hypothesized that sleep fragmentation and micro-arousals associated with snoring may act as triggers for sleep bruxism episodes via arousal-induced muscle activation [6,18]. Conversely, full-blown OSA may involve distinct pathophysiological pathways that do not consistently co-occur with bruxism. While habitual snoring was significantly associated with self-reported bruxism, this relationship may be influenced by unmeasured confounding factors such as body mass index (BMI), age, and gender, which are known to affect both snoring prevalence and sleep quality. As these variables were not fully adjusted for in the present analysis, the association should be interpreted with caution. Future studies incorporating objective sleep assessments and broader demographic controls are warranted to clarify the role of snoring in bruxism pathophysiology.

From a clinical perspective, these findings highlight the importance of a multidisciplinary diagnostic and therapeutic approach, integrating dental, psychological, and sleep medicine perspectives. Given the observed associations, future clinical investigations may explore the value of screening for TMJ symptoms and snoring in individuals with suspected bruxism, though our results alone do not support a change in practice without further validation, even when objective occlusal discrepancies are not evident. Clinical assessment protocols should include detailed a occlusal analysis, screening for TMJ dysfunction, psychosocial stressors, and sleep-related breathing disturbances. Although most of the treated participants in our study reported symptom improvement—particularly with the use of individually fabricated occlusal splints—optimal outcomes may require adjunctive behavioral and medical interventions tailored to each patient’s risk profile [16,17,28].

This study has several important limitations that warrant emphasis. First, the structured questionnaire used to assess bruxism status, occlusal interferences, and TMJ symptoms was developed specifically for this study. Although it underwent expert review and was pilot-tested for clarity, it was not subjected to formal psychometric validation or internal consistency analysis (e.g., Cronbach’s alpha), as the items addressed thematically diverse domains rather than unified constructs. Consequently, the instrument’s internal reliability and construct validity remain unknown. Second, all variables, including bruxism subtype, occlusal factors, snoring, and TMJ symptoms, were based on self-report, which introduces potential recall bias and reporting bias. Participants may have inaccurately remembered or interpreted past experiences, or reported symptoms based on prior informal diagnoses or perceptions rather than objective evaluation. This increases the likelihood of misclassification, particularly for subjective domains such as occlusal discomfort or sleep-related behaviors. Third, the use of an online snowball sampling strategy may have introduced selection bias, resulting in a sample composed predominantly of young, educated adults, thereby limiting the generalizability of findings. These methodological constraints reduce internal validity and must be considered when interpreting the observed associations. Future studies should incorporate objective clinical assessments and validated instruments to confirm and expand upon these preliminary findings. Although we followed STROBE reporting guidelines, certain elements, such as the potential for response bias due to self-selection and the lack of imputation for missing data—remain inherent limitations. The use of self-reported data to assess bruxism, TMJ-related symptoms, and occlusal interferences introduces a substantial risk of misclassification and bias. Participants may misinterpret or underreport symptoms, especially in the absence of clinical or polysomnographic confirmation. This limitation reduces the diagnostic precision of the study and should be considered when interpreting the observed associations. Future research should incorporate objective diagnostic criteria to validate self-reported findings and strengthen the evidence base.

Bruxism classification in this study relied on subjective self-perception and lay descriptions, without clinical or polysomnographic validation. This introduces a risk of misclassification, especially in differentiating between sleep and awake bruxism, and may affect the reliability of the reported associations. Stress assessment was limited to two adapted items from the PSS, which restricts the depth and validity of psychological evaluation. While this approach was chosen to minimize participant fatigue, it does not substitute for comprehensive validated instruments such as the full PSS-10 or PSS-14, which are recommended for future studies.

From a clinical perspective, these findings reinforce the multifactorial nature of bruxism and suggest potential avenues for further investigation, including the roles of occlusion, TMJ-related symptoms, and sleep-related factors. However, given the self-reported nature of the data and absence of clinical validation, recommendations regarding occlusal splints or TMJ screening should be made cautiously. Future longitudinal and clinically validated studies are needed to determine whether targeted assessments or interventions—such as occlusal therapy or TMJ evaluation—are warranted as part of a comprehensive bruxism management approach.

## 5. Conclusions

Within the constraints of its cross-sectional design and reliance on self-reported data, this study identified associations between bruxism and self-perceived occlusal interferences, TMJ-related symptoms, and habitual snoring in a Romanian adult population. While these findings support the multifactorial nature of bruxism, they should be interpreted as preliminary and hypothesis-generating rather than conclusive. The lack of objective diagnostic validation and the potential for misclassification or bias limit the ability to draw firm clinical inferences. Future research using validated instruments and clinical or polysomnographic assessments is warranted to confirm these associations and better inform multidisciplinary management strategies.

## Figures and Tables

**Table 1:** Participant Characteristics.

Characteristics	Bruxism Group (*n* = 51)	Control Group (*n* = 47)	*p*-Value
Sample size	51	47	-
Mean age (SD)	29.8 (8.5)	31.0 (9.2)	0.45 ^t^
Female (%)	28 (55%)	28 (60%)	1 ^χ2^
Male (%)	23 (45%)	19 (40%)	1 ^χ2^

Note: No statistically significant differences were found between groups in terms of age or gender distribution (*p* > 0.05), indicating comparability of demographic characteristics; t = independent samples *t*-test; χ^2^ = chi-square test.

**Table 2:** Prevalence of Occlusal Interferences and TMJ Symptoms.

Variable	Bruxism (*n* = 51)	Control (*n* = 47)	*p*-Value	Odds Ratio (95% CI)
Occlusal interference	23 (46%)	7 (14%)	0.0008	5.0 (1.9–13.0)
TMJ pain	19 (38%)	4 (8%)	0.0002	7.0 (2.2–22.2)
TMJ sounds	16 (32%)	3 (6%)	0.0014	7.4 (2.0–27.2)
Morning jaw fatigue	22 (44%)	5 (10%)	0.0001	6.8 (2.4–19.4)
Any TMJ symptom	30 (60%)	8 (16%)	<0.00001	7.5 (3.0–18.6)

Note: Bruxism was significantly associated with higher prevalence of occlusal interferences and TMJ-related symptoms (*p* < 0.001 for all comparisons), underscores a statistically significant association between bruxism-related parafunctions and self-reported TMJ symptoms.

**Table 3:** Multivariate Logistic Regression Predicting Bruxism.

Predictor	Adjusted OR	95% CI	*p*-Value
Occlusal interference	4.7	1.5–15.0	0.009
Any TMJ symptom	6.5	2.0–20.8	0.002
High daily stress	2.2	0.8–6.2	0.12
Snoring	4.1	1.3–13.2	0.016
Age (per year)	0.98	0.93–1.03	0.43
Male gender	1.1	0.4–3.0	0.85

Note: Occlusal interferences, TMJ symptoms, and snoring emerged as independent predictors of bruxism in the multivariate model. The model showed good overall fit (Hosmer–Lemeshow *p* = 0.68; Nagelkerke R^2^ = 0.58); OR = odds ratio; CI = confidence interval; *p*-values from logistic regression.

## Data Availability

The data will be available from the corresponding authors upon reasonable request.

## Supplementary Materials

The following supporting information can be downloaded at: https://www.mdpi.com/article/10.3390/jcm14165612/s1, Bruxism Questionnaire.

## Abbreviations

The following abbreviations are used in this manuscript:

AB	Awake Bruxism
CI	Confidence Interval
EMG	Electromyography
OR	Odds Ratio
OSA	Obstructive Sleep Apnea
SB	Sleep Bruxism
SD	Standard Deviation
TMJ	Temporomandibular Joint
TMD	Temporomandibular Disorders
