# Supplementary material for: Association Between Occlusal Interferences, Temporomandibular Joint Dysfunction, and Bruxism in Romanian Adults
Source: J Clin Med. 2025 Aug 8;14(16):5612. doi: 10.3390/jcm14165612 (PMC12386957; doi:10.3390/jcm14165612)
Supplement: Supplementary file 1 [file jcm-14-05612-s001.zip › jcm-3790013-supplementary.pdf]

Bruxism Questionnaire

1. Age category:

☐ <20 ☐ 20–29 ☐ 30–39 ☐ 40–49 ☐≥50

2. Gender:

☐ Male ☐ Female

3. Occupation:

☐ Student ☐ Employed ☐ Unemployed

☐ Retired ☐ Other (please specify): ____________________

4. Do you currently reside in Romania?

☐ Yes ☐ No

A. BRUXISM SCREENING

5. Have you ever been told that you grind or clench your teeth (bruxism)?

☐ Yes ☐ No ☐ Not sure

6. When does it typically occur? (select all that apply)

☐ During sleep

☐ While awake

7. Has a dentist or physician ever diagnosed you with bruxism?

☐ Yes ☐ No

B. OCCLUSAL FACTORS

8. Do you experience any of the following? (select all that apply)

☐ Morning jaw fatigue

☐ Jaw pain

☐ Tooth wear

☐ Headaches upon waking

☐ No symptoms

9. Has anyone told you that you make grinding sounds during sleep?

☐ Yes ☐ No

10. Have you received any treatment for bruxism?

☐ Yes ☐ No

11. If yes, what treatment(s) have you tried? (select all that apply)

☐ Night guard / splint

☐ Over-the-counter mouthguard

☐ Muscle relaxants or medication

☐ Stress management / therapy

☐ Other: ________________

12. Did the treatment help reduce your symptoms?

☐ Yes ☐ No

13. Has a dentist ever told you that you have a bite issue such as premature contact or occlusal interference?

☐ Yes ☐ No

14. Do you have any recent dental restorations that you feel affect your bite?

☐ Yes ☐ No

15. Do you have teeth that are misaligned or improperly positioned?

☐ Yes ☐ No

C. TMJ SYMPTOMS

16. Do you experience jaw joint pain (in front of the ear or when moving the jaw)?

☐ Yes ☐ No

17. Do you hear clicking, popping, or grinding sounds from your jaw joint?

☐ Yes ☐ No

18. Do you wake up with a tired or sore feeling in your jaw?

☐ Yes ☐ No

19. Have you ever been diagnosed with a temporomandibular disorder (TMD) by a healthcare provider?

☐ Yes ☐ No

D. PARAFUNCTIONAL HABITS

20. Do you regularly bite your nails?

☐ Yes ☐ No

21. Do you chew on objects (pens, pencils, etc.)?

☐ Yes ☐ No

22. Do you chew gum daily?

☐ Yes ☐ No

23. Do you maintain a clenched jaw posture during the day (e.g., when concentrating)?

☐ Yes ☐ No

E. SLEEP-DISORDERED BREATHING

24. Do you (or your bed partner) notice regular snoring during sleep?

☐ Yes ☐ No ☐ Not applicable

25. Have you been diagnosed with obstructive sleep apnea (OSA)?

☐ Yes ☐ No

26. Do you suspect that you may have OSA (frequent snoring, breathing pauses, daytime fatigue)?

☐ Yes ☐ No

F. PERCEIVED STRESS

Please rate your agreement with the following statements on a scale from 1 (Strongly Agree) to 5 (Strongly Disagree):

27. “I carry out daily activities in a highly tense or pressured environment.”

☐ 1 ☐ 2 ☐ 3 ☐ 4 ☐ 5

28. “My life in general is stressful.”

☐ 1 ☐ 2 ☐ 3 ☐ 4 ☐ 5

29. “My work or study environment causes me significant stress.”

☐ 1 ☐ 2 ☐ 3 ☐ 4 ☐ 5
